# The Mediating Role of Perceived Social Support Between Resilience and Anxiety 1 Year After the COVID-19 Pandemic: Disparity Between High-Risk and Low-Risk Nurses in China

**DOI:** 10.3389/fpsyt.2021.666789

**Published:** 2021-05-24

**Authors:** Tianya Hou, Qianlan Yin, Yan Xu, Jia Gao, Lian Bin, Huifen Li, Wenpeng Cai, Ying Liu, Wei Dong, Guanghui Deng, Chunyan Ni

**Affiliations:** ^1^Faculty of Psychology, Second Military Medical University, Shanghai, China; ^2^The Affiliated Suzhou Science and Technology Town Hospital of Nanjing Medical University, Suzhou, China; ^3^Changshu Hospital Affiliated to Nanjing University of Traditional Chinese Medicine, Changshu, China; ^4^Department of Psychology, Fudan University, Shanghai, China

**Keywords:** coronavirus disease 2019, anxiety, high-risk nurses, low-risk nurses, resilience, perceived social support

## Abstract

**Introduction:** One year after the Coronavirus disease 2019 (COVID-19) outbreak, China has made substantial progress in the prevention and control of the pandemic, while the epidemic situation remains grim in China since virus may easily survive with the falling temperature in winter. The present study aimed to compare the prevalence and associated factors of anxiety between high-risk and low-risk nurses 1 year after the COVID-19 outbreak, and examine the association between resilience and anxiety and its underlying mechanisms.

**Method:** Connor-Davidson Resilience scale, Perceived Social Support Scale and Generalized Anxiety Disorder Scale were administrated to 701 nurses from Jiangsu Province, China, 1 year after the COVID-19 outbreak. The mediating effect was examined by Mackinnon's four-step procedure, while the moderated mediation model was tested by Hayes PROCESS macro.

**Results:** The findings presented the prevalence of anxiety among nurses was 21.4% 1 year after the COVID-19 pandemic. High-risk nurses presented a higher prevalence of anxiety (24.5 vs. 19.3%) than low-risk nurses. Age and professional title were significantly associated with anxiety only in high-risk nurses (all *P* < 0.05). Perceived social support mediated the association between resilience and anxiety and the indirect effect was stronger for high-risk nurses than low-risk nurses.

**Conclusion:** Anxiety remains prevalent among nurses 1 year after the COVID-19 outbreak, and resilience plays a protective role against anxiety. Programs that enhance resilience and social support should be designed and special attention should be paid to nurses from high-risk units.

## Introduction

On December 30, 2019, the Coronavirus Disease 2019 (COVID-19) was first identified in Wuhan, China with unknown etiology (1). The outbreak spread rapidly throughout China and to almost every country (2). In March 2020, World Health Organization (WHO) declared COVID-19 as a global pandemic (3). Currently, the epidemic still rages in many parts of the world. As of January 29, 2021, the number of confirmed COVID-19 cases worldwide has reached 100 million, with more than 2 million confirmed deaths reported (4). Many countries are facing the “second wave” of the COVID-19 outbreak since it is easier for the novel coronavirus to survive with the falling temperature in winter. The epidemic situation is still grim in China and scattered COVID-19 outbreaks are unavoidable (5). A slightly increasing trend in infections has been reported in China during the past month. As of the end of January 2021, a total of 1614 patients were diagnosed with COVID-19 in China (6). To curb the pandemic in this winter, provincial government of Jiangsu has issued notifications to expand nucleic acid testing to a larger scale and enhance the frequency of regular testing (7). There is no doubt that these measures have placed a great burden on the healthcare system. The availability of health care workers is vital in controlling the pandemic.

In the face of the continuing crisis, nurses, as the largest part of health care workers (8), are crucial to the pandemic response and care. Nurses are inevitably confronted with a broad range of stressors such as overwhelming workload caused by the high demand on the strained system, shift duties, work-family conflict and so on (9), which makes them, especially high-risk nurses, more susceptible to poor mental health (10). According to the previous literature, health care workers from high-risk working departments had a higher rate of mental problems than those from low-risk working departments during the COVID-19 outbreak (11). McAlonan et al. (12) pointed out that although no significant differences in the stress level were found between high-risk and low-risk health care workers at the initial stage of Severe Acute Respiratory Syndrome (SARS) outbreak, high-risk nurses exhibited higher levels of stress than low-risk nurses 1 year after the SARS outbreak. Psychological problems negatively affect cognitive functioning, including attention, memory, risk perception and so on, which would further impair work performance (13). Under the current circumstance, it is of great importance to pay special attention to the mental health of nurses as a way of avoiding unnecessary attrition of hospital workforce. Previous literature regarding the psychological impact of COVID-19 on health professionals suggested the heavy mental toll of COVID-19 outbreak in nurses, with anxiety being frequently observed (14, 15). To date, existing literature has rarely investigated the prevalence of anxiety under the current epidemic situation and the differences in the associated factors of anxiety between high-risk and low-risk nurses. Thus, there is an urgent need to explore the impact of COVID-19 on nurses' mental health and potential influential factors of anxiety 1 year after the pandemic.

Resilience is defined as the capacity to successfully thrive and adapt despite encountering adversity (16). Resilience could decrease the negative impact of stressful events on mental health (17). Resilient individuals presented less severe psychological problems and recovered more quickly from the adversity (18). Previous studies have consistently proved the negative association between resilience and anxiety both cross-sectionally and longitudinally (18–20). Thus, we expected that resilience exerted a protective effect on the anxiety of nurses 1 year after the COVID-19 outbreak. Social support is another common protective factor against psychological difficulties. According to the stress-buffering hypothesis proposed by Cohen and Wills (21), social support could act as a buffer against negative outcomes of stress through enhancing the experience of self-esteem and self-efficacy. Perceived social support is the individuals' subjective perception of the amount and quality of supportive resources received from their social networks. Perceived social support is positively correlated with mental well-being and helps individuals cope with stressful events (22, 23). Several recent studies have investigated the role of perceived social support pertaining to anxiety amid the COVID-19 pandemic. Young adults with lower levels of social support had a higher risk of experiencing anxiety symptoms than those with higher levels of social support during the first several weeks of COVID-19 pandemic (24). In a study performed on frontline nurses during the COVID-19 pandemic, nurses with high social support were less likely to suffer from anxiety (25). Furthermore, it is well-documented that resilience as an internal factor and perceived social support as an external factor are highly correlated (26, 27). Resilient people tend to have strong social networks, which are the great sources of support when they are facing the difficulties. Additionally, resilient individuals had more positive perceptions toward current situation, which indicates resilient people would report higher levels of perceived social support than non-resilient people who have the same actual social support (28). Thus, we hypothesized that social support might be the possible pathway through which resilience influences anxiety of nurses 1 year after the COVID-19 pandemic.

Although resilience and social support might influence anxiety of nurses, not all nurses with lower levels of resilience or social support experience more anxiety symptoms. Hence, it is vital to investigate the influential factor that might moderate the associations of resilience and social support with anxiety among nurses 1 year after the COVID-19. As mentioned above (12), high-risk nurses might be more susceptible to develop anxiety 1 year after the outbreak. Thus, we speculated that high-risk nurses might experience higher levels of anxiety than low-risk nurses 1 year after the COVID-19 outbreak and the pathway through which anxiety could be mitigated would be strengthened among high-risk nurses. Specifically, the associations of resilience and social support with anxiety would be stronger for nurses from high-risk units than those from low-risk units 1 year after the COVID-19 pandemic.

**Figure 1:**
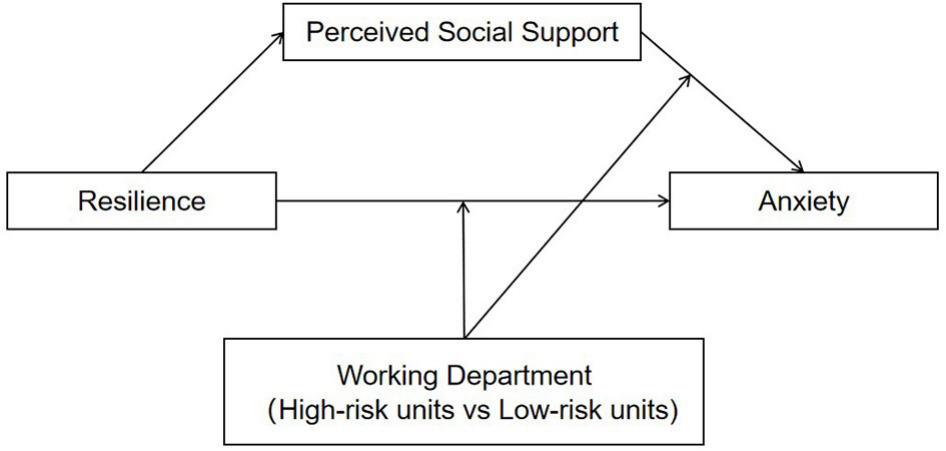
Conceptual model.

To date, no study has investigated the sustained psychological impact of COVID-19 on nurses and the potential mechanisms underlying the association between resilience and anxiety 1 year after the COVID-19 pandemic, especially differentiating high-risk and low-risk nurses. Therefore, the present study was initiated to investigate the prevalence of anxiety among nurses and compare the associated factors of anxiety between high-risk and low-risk nurses 1 year after the COVID-19 outbreak. Moreover, this study examined the mediating role of social support between resilience and anxiety, and whether the association between resilience and anxiety through perceived social support differed between high-risk and low-risk nurses. A moderated mediation model is proposed to address the hypotheses perceived social support might mediate the effect of resilience on anxiety and the direct and/or indirect (perceived social support—anxiety path) effect of resilience on anxiety might be stronger for high-risk nurses than low-risk nurses 1 year after the COVID-19 pandemic (see Figure 1).

## Method

### Participants and Procedures

This cross-sectional study was conducted in early January, 2021, 1 year after the outbreak of COVID-19 pandemic. Random cluster sampling was utilized to enroll nurses from 5 local hospitals in Jiangsu Province China. A total of 707 nurses voluntarily completed the questionnaires. The inclusion criteria were as follows: (a) working in the hospital from the height of the COVID-19 outbreak to 1 year after, (b) no dyslexia, and (c) age > 18 years. The exclusion criterion was that respondents reported a history of mental illnesses. Finally, 701 participants were included in the analysis.

Ethical approval was obtained from the Second Military Medical University before the initiation of the research project. Prior to the online survey, informed written consent was given by all participants. Participants were assured their responses were anonymous and confidential. Participants were free to withdraw at any time without penalty.

### Measures

#### Demographics

In the present study, demographic information included age, gender, marital status, educational level, years of working, professional title, and working department. Age was divided into two intervals (younger group and middle-aged group). Marital status was categorized into married and unmarried (single, divorced, and widowed). Educational level was classified into two levels (high school or under and college or above). Years of working was set as a binary variable (5 years or less and more than 5 years). Professional title was divided into three levels: junior, intermediate and senior. Working departments were grouped into high-risk and low-risk units. Nurses from fever clinics, COVID-19 medical unit and emergence department were considered high-risk, while the others were identified as low-risk nurses (29). Fever clinics are specialist clinics that are responsible for the identification of COVID-19 infections. COVID-19 medical units are designated to treat COVID-19 patients. Nurses from emergency department work as the initial hospital caregivers for the badly ill patients with confirmed or suspected COVID-19. Thus, compared with other departments, nurses from fever clinics, COVID-19 medical unit and emergence department have a higher chance of contacting COVID-19 patients.

#### Resilience

The 25-item Connor-Davidson Resilience scale (CD-RISC) was employed to measure resilience (30). The items used a 5-point Likert scale ranging from 0 (never) to 4 (very often). The Chinese version of CD-RISC supports a three-factor structure including tenacity (i.e., “when things look hopeless, I don't give up”), strength (i.e., “able to adapt to change”), and optimism (i.e., “can deal with whatever comes”). The total scores were the sum of the 25 items and ranged from 0 to 100, with higher scores denoting greater resilience. The scale has demonstrated strong reliability and validity (31, 32). In the current study, the Cronbach's alpha was 0.984 for the total scale, 0.976 for tenacity subscale, 0.960 for strength subscale and 0.854 for optimism subscale.

#### Perceived Social Support

The Chinese version of Perceived Social Support Scale (PSSS) was utilized to measure the perception of social support quality (33). The scale consists of three subscales indexing the sources of social support from friends (i.e., “I can talk about my problems with my friends”), family (e.g., “I get the emotional help and support I need from my family”) and significant others (e.g., “I have a special person who is a real source of comfort to me”), respectively. Participants were required to rate their agreement with each item using a 7-point Likert scale from 1 (very strongly disagree) to 7 (very strongly agree). Items were summed to yield total scores ranging from 12 to 84. The scale has been widely used with excellent psychometric properties (34, 35). In the present study, the Cronbach's alpha was 0.968 for the overall scale, 0.949 for the friend subscale, 0.938 for the family subscale and 0.908 for the significant others subscale.

#### Anxiety

The 7-item Generalized Anxiety Disorder Scale (GAD-7) was used to measure anxiety over the past 2 weeks (36). Participants were asked to rate each item on a 4-point Likert scale ranging from 1 (never) to 3 (nearly every day). Item ratings were summed to obtain total scores ranging from 0 to 21, with higher scores representing more severe symptoms of anxiety. The cut-off point for identifying the symptoms of anxiety was 7 (37). The scale has been widely used and adequate psychometric properties have been reported for the Chinese version of GAD-7 (10, 37). The Cronbach's alpha for GAD-7 was 0.952 in the present study.

### Statistical Analysis

Firstly, Harman single factor test was employed to test common method bias and descriptive analyses were conducted to describe demographic characteristics stratified by working department. Secondly, linear regressions were used to calculate univariate associations between demographic characteristics and anxiety stratified by working department. Thirdly, Pearson's correlation analyses were calculated to explore the bivariate correlations between the variables of interest. Fourthly, Mackinnon's four-step procedure was employed to examine the mediation effect (38). Four conditions should be met to establish the mediating effect: (1) a significant association of resilience with anxiety; (2) a significant association between resilience and perceived social support; (3) a significant association between perceived social support and anxiety after controlling for resilience; (4) a significant coefficient for the indirect path between resilience and anxiety *via* perceived social support. The last condition was determined by the bias-corrected percentile bootstrap method, which has been widely employed to construct the sampling distribution of a statistic. Bootstrap sampling is a statistical process that could resample a single dataset to create a distribution of datasets and bootstrap sampling distributions are created by sampling repeatedly with replacement from the original dataset (39). In the present study, bootstrap method produced 95% bias- corrected confidence intervals (CIs) based on 5000 resamples of the data. If the 95% CI does not include zero, the mediating effect would be established. Compared with the traditional causal steps approach, bootstrapping method is better in statistical power and type I error (40). The parameters for the mediation effect were estimated by Hayes (41) PROCESS macro (Model 4).

Finally, we utilized Hayes (41) PROCESS macro (Model 15) to examine the moderated mediation effect. SPSS macro PROCESS, a computational tool, has been widely used to examine complex models with both mediating and moderating variables (42, 43). A set of models have been defined by a model number and preprogrammed into PROCESS. Researchers could choose a model corresponding to the diagram they estimate (44). The 95% CI that excluded zero indicated the establishment of the moderated mediation model. Simple slope test was performed for further analysis.

Age, gender, marital status, educational level, years of working and professional title were included in all models as covariates and all variables were standardized. All statistical analyses were performed using SPSS 25.0 and two-tailed *P*-values < 0.05 were considered statistically significant.

## Results

### Common Method Bias Test

The research data were collected using a self-report survey, which indicated a possibility of a common method bias problem (45). The Harman single factor test was conducted to test common method bias (46, 47). The KMO value was 0.963 (*p* < 0.001), suggesting the research data were suitable for factor analysis. Exploratory factor analysis presented that there were 20 factors with eigenvalues more than 1 and the interpretation rate of the first factor was 25.335%, which did not reach the reference value of 40%. Hence, the results revealed that there was no serious common method bias problem in this research.

### Demographic Characteristics Stratified by Working Department

The demographic characteristics stratified by working department are presented in Table 1. Of the 701 respondents, 265 (37.8%) worked in the high-risk unit. Most of the participants were female [653 (93.2%)], aged below 30 years [391 (55.8%)] and married [455 (64.9%)], had an educational level of college or above [691 (98.6%)], worked more than 5 years [382 (54.5%)] and obtained a junior technical title [491 (70.0%)].

**Table 1:** The demographic characteristics of high-risk and low-risk nurses (*N* = 701).

**Variables**	**Total sample (*****n*** **=** **701)**		**High-risk nurses (*****n*** **=** **265)**		**Low-risk nurses (*****n*** **=** **436)**	
	***n***	**Ratio (%)**	***n***	**Ratio (%)**	***n***	**Ratio (%)**
**Gender**						
Male	48	6.8	17	6.4	31	7.1
Female	653	93.2	248	93.6	405	92.9
**Age**						
Younger group (18–30 years)	391	55.8	153	57.7	238	54.6
Middle-aged group (30–54 years)	310	44.2	112	42.3	198	45.4
**Marital status**						
Unmarried	246	35.1	101	38.1	145	33.3
Married	455	64.9	164	61.9	291	66.7
**Educational level**						
High school or under	10	1.4	6	2.3	4	0.9
College or above	691	98.6	259	97.7	432	99.1
**Years of working**						
≤ 5 years	319	45.5	133	50.2	186	42.7
>5 years	382	54.5	132	49.8	250	57.3
**Technical title**						
Junior	491	70.0	198	74.7	293	67.2
Intermediate	179	25.5	57	21.5	122	28.0
Senior	31	4.4	10	3.8	21	4.8

### Associations of Demographic Characteristics With Anxiety

The prevalence of anxiety among nurses 1 year after the COVID-19 pandemic was 21.4%. The prevalence of anxiety in high-risk nurses was 24.9%, whereas the rate of anxiety in low-risk nurses was 19.3%. In the univariate linear regression model, high-risk nurses presented a significantly higher level of anxiety than low-risk nurses [β = −0.102, 95% CI = (−1.544, −0.250)]. Female nurses reported more anxiety than males only in high-risk group [β = 0.137, 95% CI = (0.335, 5.021)]. Across the overall sample, middle-aged nurses experienced more symptoms of anxiety [β = 0.103, 95% CI = (0.252, 1.515)]. However, the difference was not significant in low-risk group. In the overall sample, nurses with intermediate professional titles had more anxiety than those with junior professional titles [β = 0.083, 95% CI = (0.084, 1.537)]. Nonetheless, the disparity disappeared in low-risk nurses (see Table 2).

**Table 2:** Association of demographic characteristics with anxiety stratified by working department.

**Variables**	**Total sample (*n* = 701)**	**High-risk nurses (*n* = 265)**	**Low-risk nurses (*n* =4 36)**
	**β (95% CI)**	**β (95% CI)**	**β (95% CI)**
**Gender**			
Male	Reference	Reference	Reference
Female	0.06 (−0.246, 2.247)	0.137^*^ (0.335, 5.021)	0.003 (−1.376, 1.453)
**Age**			
Younger group (18 ~30 years)	Reference	Reference	Reference
Middle-aged group (30–54 years)	0.103^***^ (0.252, 1.515)	0.158^*^ (0.364, 2.681)	0.07 (−0.183, 1.274)
**Marital status**			
Unmarried	Reference	Reference	Reference
Married	0.025 (−0.435, 0.886)	−0.007 (−1.265, 1.122)	0.06 (−0.278, 1.262)
**Educational level**			
High school or under	Reference	Reference	Reference
College or above	−0.041 (−4.142, 1.172)	−0.045 (−5.33, 2.453)	−0.026 (−4.874, 2.749)
**Years of working**			
≤ 5 years	Reference	Reference	Reference
>5 years	0.074 (−0.004, 1.259)	0.094 (−0.255, 2.053)	0.073 (−0.164, 1.302)
**Technical title**			
Junior	Reference	Reference	Reference
Intermediate	0.083^*^ (0.084, 1.537)	0.159^*^ (0.446, 3.244)	0.046 (−0.421, 1.214)
Senior	0.055 (−0.4, 2.681)	0.091 (−0.727, 5.305)	0.037 (−1.054, 2.374)

* *P < 0.05.*

*** *P < 0.001*.

### Bivariate Analysis

Table 3 shows the descriptive statistics and correlations among the variables of interest. The results revealed that resilience and its three subscales were all significantly and positively correlated with perceived social support and its three subscales (all *P* < 0.001). Anxiety was significantly and negatively related to resilience, perceived social support and their subscales (all *P* < 0.001).

**Table 3:** Descriptive statistics and correlation analysis among the variables of interest.

	**Mean (SD)**	**1**	**2**	**3**	**4**	**5**	**6**	**7**	**8**	**9**
1. Working department	1.62 (0.49)	1								
2. Tenacity subscale of CD-RISC	30.54 (11.35)	0.063	1							
3. Strength subscale of CD-RISC	19.98 (6.95)	0.064	0.938^***^	1						
4. Optimism subscale of CD-RISC	9.24 (3.52)	0.058	0.831^***^	0.888^***^	1					
5. Resilience (CD-RISC)	59.76 (21.15)	0.064	0.983^***^	0.980^***^	0.904^***^	1				
6. Friend subscale of PSSS	20.55 (4.68)	0.037	0.394^***^	0.412^***^	0.356^***^	0.406^***^	1			
7. Family subscale of PSSS	20.73 (4.71)	0.066	0.382^***^	0.390^***^	0.354^***^	0.392^***^	0.845^***^	1		
8. Significant others subscale of PSSS	19.71 (4.63)	0.015	0.397^***^	0.414^***^	0.356^***^	0.408^***^	0.875^***^	0.803^***^	1	
9. Perceived social support (PSSS)	61.00 (13.26)	0.042	0.414^***^	0.428^***^	0.376^***^	0.425^***^	0.959^***^	0.934^***^	0.944^***^	1
10. Anxiety (GAD-7)	3.54 (4.25)	−0.102^**^	−0.253^***^	−0.273^***^	−0.199^***^	−0.259^***^	−0.375^***^	−0.355^***^	−0.381^***^	−0.391^***^

** *P < 0.01.*

*** *P < 0.001*.

### Testing the Mediating Role of Perceived Social Support

As noted, we predicted perceived social support would mediate the association between resilience and anxiety among nurses. MacKinnon's (38) four-step procedure was performed to examine the mediating effect (see Table 4). Firstly, resilience was significantly related to anxiety (β = −0.253, *P* < 0.001) (see Model 1 in Table 4). Secondly, resilience was significantly associated with perceived social support (β = 0.421, *P* < 0.001) (see Model 2 in Table 4). Thirdly, perceived social support was significantly associated with anxiety when resilience was controlled (β = −0.348, *P* < 0.001) (see Model 3 in Table 4). Finally, the biased-corrected percentile bootstrap method indicated the indirect effect of resilience on anxiety *via* perceived social support was significant [ab = −0.147, SE = 0.025, 95% CI = (−0.199, −0.101)]. The ratio of the indirect effect of the total effect was 57.9%. Thus, all four conditions for the mediation effect have been satisfied and perceived social support mediated the relation between resilience and anxiety among nurses 1 year after the outbreak of COVID-19.

**Table 4:** Mediation analysis (*N* = 701).

	**Model 1 (anxiety)**		**Model 2 (perceived social support)**		**Model 3 (anxiety)**		**Indirect effect of perceived social support**				
	**β**	***t***	**β**	***t***	**β**	***t***		**Indirect effect**	**SE**	**LLCI**	**ULCI**
Resilience	−0.253^***^	−6.922	0.421^***^	12.248	−0.107^**^	−2.795	Perceived social support	−0.147	0.025	−0.199	−0.101
Perceived social support					−0.348^***^	−9.098					
$R_{\text{adj}}^{2}$	0.079		0.187		0.177						
*F*	7.388^***^		19.838^***^		16.539^***^						

*All models are adjusted for age, gender, marital status, educational level, years of working, and professional title.*

** *P < 0.01.*

*** *P < 0.001*.

### Testing for Moderated Mediation Effect

The study expected the working department would moderate the direct and indirect (the second stage of the mediating path: perceived social support—anxiety) effects of resilience on anxiety. The results showed the interaction of perceived social support and working department had a significant effect on anxiety (β = 0.169, *P* < 0.05), suggesting the association between perceived social support and anxiety was moderated by working department (see Table 5). Thus, the moderated mediation was established in our study since the second stage of the mediation effect was moderated by working department (41).

**Table 5:** Testing the moderated mediation effect (*N* = 701).

	**β**	***SE***	**LLCI**	**ULCI**
**Mediator variable model (outcome: perceived social support)**				
Resilience	0.421^***^	0.034	0.353	0.488
**Dependent variable model (outcome: anxiety)**				
Resilience	−0.122^*^	0.060	−0.240	−0.003
Perceived social support	−0.454^***^	0.060	−0.573	−0.336
Working department	−0.222^**^	0.071	−0.362	−0.083
Resilience ^*^ working department	0.023	0.078	−0.131	0.176
Perceived social support ^*^ working department	0.169^*^	0.078	0.015	0.322
**Conditional indirect effect analysis**				
High-risk unit	−0.191	0.038	−0.270	−0.120
Low-risk unit	−0.120	0.270	−0.176	−0.071
Index of moderated mediation	0.071	0.041	−0.007	0.153

*All models are adjusted for age, gender, marital status, educational level, years of working, and professional title.*

* *P < 0.05.*

** *P < 0.01.*

*** *P < 0.001*.

**Figure 2:**
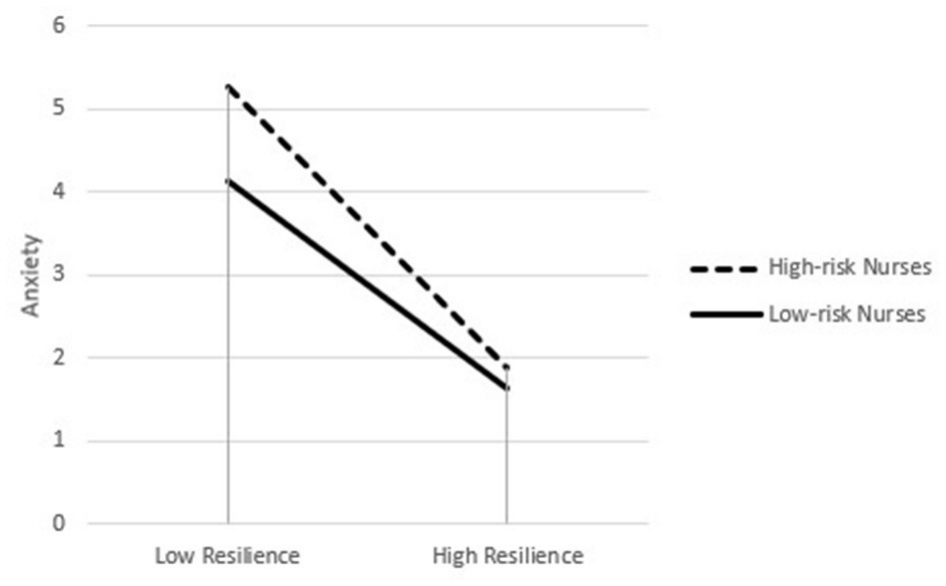
Working department as a moderator of the association between resilience and anxiety.

Simple slope analysis revealed perceived social support was significantly and negatively related to anxiety for high-risk nurses (β_*simple*_ = −0.285, *P* < 0.001), while for low-risk nurses, the association between perceived social support and anxiety became insignificant (β_*simple*_ = −0.117, *P* = 0.315). For descriptive purpose, the current study plotted the association between perceived social support and anxiety, separately for high-risk and low-risk nurses (see Figure 2).

Table 5 also presented the indirect effect was moderated by working department. Specifically, the indirect effect of resilience on anxiety through perceived social support was stronger for high-risk nurses [β= −0.191, 95% CI = (−0.270, −0.120)] than low-risk nurses [β = −0.120, 95% CI = (−0.176, −0.071)].

## Discussion

Our results revealed that the overall prevalence of anxiety among nurses was 21.4% 1 year after the COVID-19 pandemic, which suggests that more than one-fifth of the nurses suffer from anxiety. This is lower than the one reported by a meta-analysis (15) during the peak period of the COVID-19 pandemic (27%). However, our result is 3 times higher when compared with the reported prevalence of anxiety across the globe (7.3%) in non-epidemic period (48). This indicated, despite the prevalence of anxiety diminished from the peak of the COVID-19 outbreak in 2020 to 1 year after, nurses still experience higher levels of anxiety. The findings also showed the prevalence found in high-risk nurses (24.5%) was higher than that in low-risk nurses (19.3%) 1 year after the outbreak of COVID-19. This is consistent with previous literature, suggesting the ongoing adverse mental health impact is stronger in high-risk nurses 1 year after the SARS outbreak (12). More attentions should still be paid to the anxiety of nurses even 1 year after the pandemic, which might exert the detrimental effect on the safety of patients and prevention and control of COVID-19 (49).

Our study presented that gender was significantly associated with anxiety only in high-risk nurses. However, it is important to note that the sample in the present study is very skewed with regard to the gender variable. The gender-driven finding could be totally biased since 93.2% of the participants were female. Our study also found that age and professional title were significantly associated with anxiety only in high-risk nurses. Compared with younger high-risk nurses, anxiety appeared to reach a higher level among middle-aged nurses. This might be due to the fact that middle-aged nurses were more likely to take responsibilities to raise both elderly parents and young children. Thus, apart from the occupational stress, middle-aged high-risk nurses also faced the greater pressure to raise families (50), which might account for the age difference in anxiety. It is noteworthy to mention high-risk nurses with intermediate professional titles presented the higher level of anxiety than those with junior professional titles. Compared with the inexperienced and inadequately-trained junior nurses, nurses with intermediate professional titles not only need to implement the decisions made by senior nurses with physical and mental labor, but also need to take more responsibilities to guide and help junior nurses to complete the work.

As expected, perceived social support partially mediated the relationship between resilience and anxiety among nurses 1 year after the outbreak, which is consistent with the existing literature regarding the mediating role of social support in the resilience—psychological problems path (51–53). Moreover, the moderated mediation analysis found that working department moderated the indirect effect of resilience on anxiety *via* perceived social support among nurses, which partially supported our hypothesis. Specifically, nurses from high-risk working units showed stronger association between perceived social support and anxiety 1 year after the COVID-19 pandemic. This study adds to the existing literature by combining internal (resilience) and external (social support) resources and the environmental characteristic (working department) to explore anxiety among nurses 1 year after the outbreak. These findings have profound theoretical and practical implications. This study provided a theoretically grounded framework for a more in-depth understanding of the underlying mechanisms behind the association between resilience and anxiety among nurses. Practically, the present study informed public health policies of the potential preventive measures of anxiety for nurses 1 year after the outbreak. To help prevent anxiety 1 year after the COVID-19 outbreak, programs that enhance resilience and social support should be designed and special attention should be paid to nurses from high-risk units. Additionally, policies on hospital infection-control measures should be designed and implemented to reduce the risk of being infected in the hospital. Before each shift, hospital staff should be screened to check out the signs of COVID-19 infection, such as fever and cough. All staff should be required to wear personal protective equipment and practice social distancing. Emergency infection protocols should be instituted by hospital to contain COVID-19. Clinical inpatient and outpatient services should be adjusted and all non-essential visits should be suspended (54).

### Limitation

Some limitations should be addressed when interpreting the results. Firstly, the current study utilized a cross-sectional design, which is insufficient to infer the temporal sequence among variables. We are unable to tease apart the causal relationship. Longitudinal or experimental research should be conducted in the future to verify the findings. Second, the present study utilized self-reported scales, which might bias the association among variables. In the future studies, multi-informant approaches are suggested to collect data. Thirdly, nurses analyzed in our study were only from Jiangsu Province, limiting the generalization of the findings. Further studies are needed to recruit participants from a broad range of geographic regions. Fourthly, we observed a very skewed gender distribution in our sample, with 93.2% of the participants being females. The gender-related findings might be totally biased. Further study should be conducted to replicate our findings in a gender-balanced sample. Fifthly, anxiety could be affected by a variety of factors. The model in our study could just account for a part of the variance. Therefore, a more integrated model of anxiety of nurses is recommended for future research. Finally, apart from anxiety, depression is another common mental disorder that needs to be addressed. Further study should investigate the prevalence of and factors associated with depression 1 year after the COVID-19 pandemic.

To the best of our knowledge, this is the first study to report the prevalence of anxiety among nurses 1 year after the COVID-19 pandemic, and examine the relationship between resilience and anxiety through a moderated mediation model. Anxiety remains prevalent (21.4%) in the nursing workforce 1 year after the COVID-19 outbreak. Special attentions should be paid to nurses from high-risk units, especially those who were middle-aged and had intermediate professional titles. The resilience-based psychological program such as self-enabled distraction and relieving stress from exercise should be designed to prevent and reduce anxiety of nurses (55). Our findings also suggested non-resilient nurses, especially those working in the high-risk unit, could benefit from interventions aiming at the enhancement of perceived social support, including expanding social networks, increasing the quality of social support and so on, 1 year after the COVID-19 pandemic (56).

## Data Availability Statement

The raw data supporting the conclusions of this article will be made available by the authors, without undue reservation.

## Ethics Statement

The studies involving human participants were reviewed and approved by Second Military Medical University. The patients/participants provided their written informed consent to participate in this study.

## Author Contributions

TH, QY, YX, and JG contributed to the writing of this article and the statistical analysis. CN, GD, and WD leaded the whole study, including carrying out this study, and putting forward the study. LB, HL, WC, and YL contributed to the data collection and statistical analysis. All authors contributed to editing the manuscript and have approved the final manuscript.

## Conflict of Interest

The authors declare that the research was conducted in the absence of any commercial or financial relationships that could be construed as a potential conflict of interest.
